# Application and safety analysis of paravertebral block with dezocine and ropivacaine in video-assisted thoracoscopic surgery for lung cancer

**DOI:** 10.3389/fmed.2025.1723385

**Published:** 2026-01-27

**Authors:** Dengxue Gan, Faju Wan, Hui Liu, Yong Wu

**Affiliations:** 1 Department of Cardiothoracic Vascular, Fushun People’s Hospital, Zigong, Sichuan, China; 2 Department of Anesthesiology, The Third Affiliated Hospital of Chongqing Medical University, Chongqing, China; 3 Department of Anesthesiology, The Affiliated Hospital, Southwest Medical University, Luzhou, Sichuan, China

**Keywords:** dezocine, non-small cell lung cancer, postoperative adverse reactions, ropivacaine, thoracic paravertebral block, video-assisted thoracoscopic surgery

## Abstract

**Objective:**

This study aimed to analyze the application and safety of different doses of dezocine combined with ropivacaine for thoracic paravertebral block (TPVB) in video-assisted thoracoscopic surgery (VATS) for lung cancer.

**Methods:**

A total of 192 patients with non-small cell lung cancer undergoing VATS were prospectively enrolled and randomly allocated into two groups using a random number table method: L-dose (0.1 mg⋅kg^–1^ dezocine + 0.375% ropivacaine for TPVB before general anesthesia induction, *n* = 96) and H-dose (0.15 mg⋅kg^–1^ dezocine + 0.375% ropivacaine for TPVB before general anesthesia induction, *n* = 96). Clinical baseline data, surgical time and recovery-related time (spontaneous breathing recovery, awakening, and extubation), analgesic use within 12 h, pain scores at 1, 6, 12, and 24 h (VAS, resting and active states), serum inflammatory markers [CRP, TNF-α, procalcitonin (PCT)], and adverse events were recorded.

**Results:**

Relative to the H-dose group, the L-dose group showed faster recovery of breathing, awakening, and extubation (all *P* < 0.001). The H-dose group required fewer analgesic pump uses and rescue analgesia within 12 h (16.67% vs. 46.88%, *P* < 0.001). VAS scores increased postoperatively, peaked within 12 h, and declined at 24 h; at all time points, scores were lower in the H-dose group (all *P* < 0.05). Both groups showed transient increases in CRP, TNF-α, and PCT at 24 h, followed by declines after drain removal (all *P* < 0.01). The overall incidence of adverse events did not differ markedly between the L-dose and H-dose groups (6.25% vs. 8.33%, *P* > 0.05).

**Conclusion:**

When used in TPVB for VATS, different doses of dezocine combined with ropivacaine offer distinct short-term postoperative benefits: the 0.1 mg⋅kg^–1^ dezocine results in faster postoperative recovery, whereas the 0.15 mg⋅kg^–1^ dezocine demonstrates superior postoperative analgesic efficacy and inflammation suppression. Both doses exhibited high safety profiles, providing certain references for the selection of clinical anesthesia protocols.

## Introduction

Over the past decade, substantial advances have reshaped the management of non-small cell lung cancer (NSCLC), with progress spanning screening, diagnostics, and treatment modalities (1). Despite innovations in surgical techniques, chemotherapy, radiotherapy, alongside targeted therapy, the 5-year survival rate for NSCLC patients continues to lag at below 50% (2). Since the first lobectomy performed in the early 1990s, video-assisted thoracoscopic surgery (VATS) has become widely utilized in thoracic surgery and is now the standard surgical approach for early-stage NSCLC (3). Compared with conventional thoracotomy, VATS offers multiple advantages as a minimally invasive technique, such as reduced blood loss during surgery, less pain afterward, fewer complications, quicker recovery, shorter hospitalization, and lower mortality (4). Recent studies have confirmed that VATS can improve overall survival, shorten hospital stays, and demonstrate superior perioperative outcomes and long-term survival advantages compared to thoracotomy (5, 6). Although VATS is less invasive than thoracotomy, patients often experience moderate postoperative pain and inflammatory responses, which may delay recovery (7). Therefore, identifying effective methods to enhance postoperative analgesia and promote recovery in patients undergoing VATS for lung cancer is crucial.

Thoracic paravertebral block (TPVB) is a common postoperative analgesic method for patients undergoing thoracic surgery, significantly alleviating pain from surgical incisions (8). Currently, long-acting local anesthetic ropivacaine is commonly used in clinical TPVBs to prolong analgesic effects (8, 9). Interestingly, experimental studies have shown that ropivacaine may also influence the progression of non-small cell lung cancer through molecular pathways (10). However, the analgesic effect of ropivacaine in TPVBs typically lasts only about 6 h, which is insufficient for effective relief of postoperative pain in thoracic surgery (8, 11).

Existing data indicate that dezocine combined with ropivacaine infiltration anesthesia can effectively shorten the anesthesia recovery time in patients undergoing open liver resection, suppress the release of pain factors, reduce postoperative stress responses and immune function fluctuations, and help lower postoperative pain levels (7). Dezocine is a synthetic potent analgesic with high lipophilicity, whose pharmacological properties lie in its ability to simultaneously activate κ receptors and partially antagonize μ receptors (12). Compared to traditional pure μ receptor agonists, this unique receptor mechanism enables it to provide effective analgesia while reducing the risk of related adverse reactions and exhibiting potential immunomodulatory functions (12–14). Based on these characteristics, dezocine has been widely used in clinical perioperative pain management and is often combined with other analgesic drugs to enhance analgesic effects or improve medication safety (12–14). Additionally, the combined application of dezocine and dexmedetomidine as adjuvants to general anesthesia can improve inflammatory factor levels in patients undergoing lung cancer surgery (15).

Given the above research background, although the combined analgesic effect of dezocine and ropivacaine has shown advantages in some surgeries, the impact of different doses of dezocine via the TPVB route on analgesic efficacy, inflammatory immune regulation, and safety in the specific context of VATS for lung cancer has not been systematically elucidated. Therefore, this study aims to systematically evaluate the analgesic effects, safety, and impact on perioperative inflammatory responses of different doses of dezocine combined with ropivacaine for TPVB in VATS for lung cancer, with the goal of providing evidence for optimizing postoperative analgesic regimens in clinical practice.

## Materials and methods

### Ethnic statement

Approval was attained from the institutional ethics committee of The Affiliated Hospital, Southwest Medical University, and all participants provided written informed consent prior to sample collection.

### Study subjects

**FIGURE 1:**
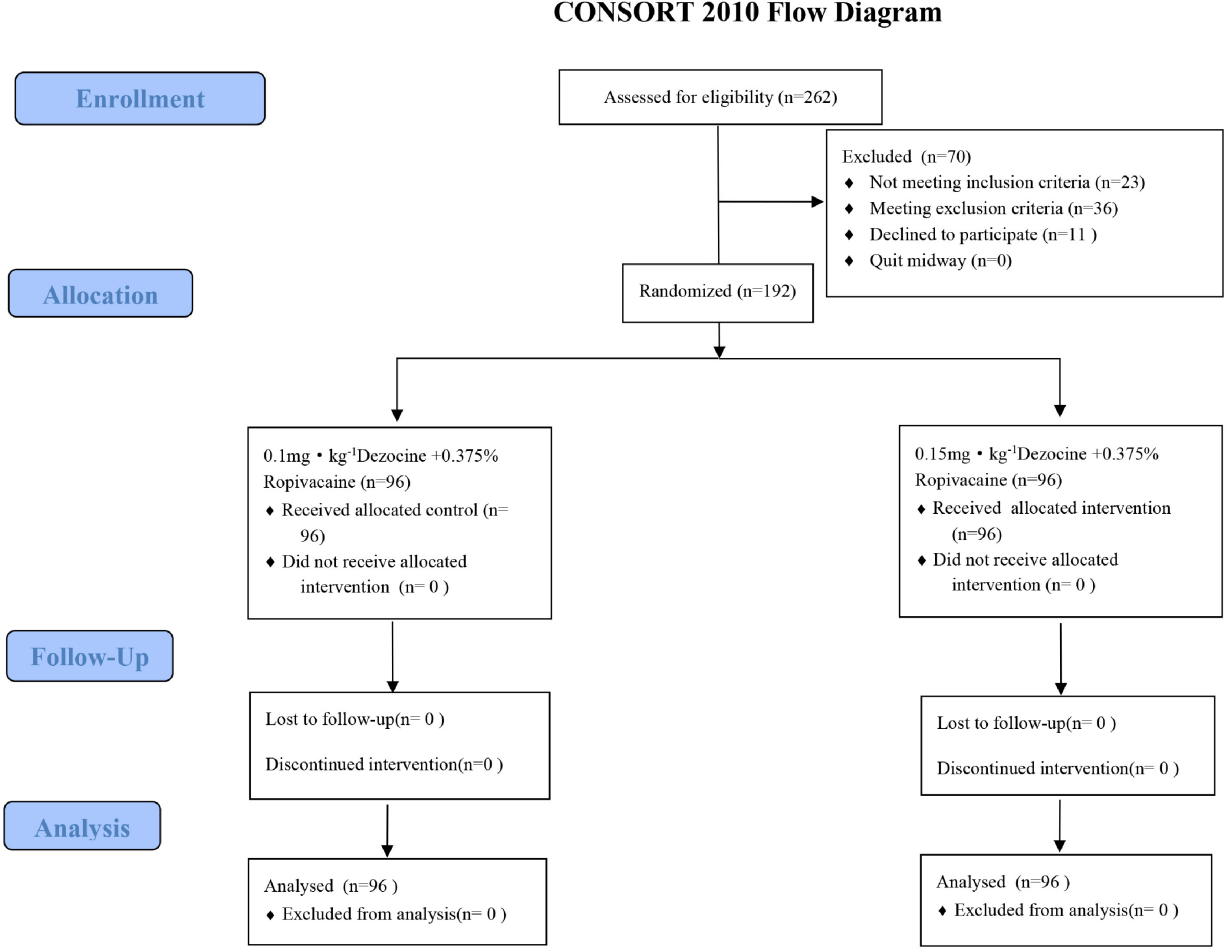
Flow chart.

Between June 2022 and December 2024, 262 patients with NSCLC scheduled for VATS at our hospital were prospectively screened. Of these, 23 did not meet the inclusion criteria, 36 were excluded according to predefined criteria, and 11 either lacked complete data or declined participation. Ultimately, 192 eligible patients were included. Patients were grouped and studied according to the random number table method, with two groups established: the L-dose group (receiving 0.1 mg⋅kg^1^ dezocine combined with 0.375% ropivacaine, 20 mL, *n* = 96) and the H-dose group (receiving 0.15 mg⋅kg^–1^ dezocine with 0.375% ropivacaine, 20 mL, *n* = 96). No participants withdrew during the trial. A detailed flowchart is provided in Figure 1.

### Inclusion and exclusion criteria

Eligible participants met the following criteria: (1) confirmed diagnosis of NSCLC by pathology, with disease staged as ≤ II according to tumor-node-metastasis (TNM) classification (16), and had a TNM stage of ≤ II; (2) indications for VATS surgery; (3) age between 20 and 60 years; (4) New York Heart Association (NYHA) functional class I–II; (5) American Society of Anesthesiologists (ASA) physical status I–II; and (6) signed written informed consent.

Exclusion criteria were: (1) TNM stage ≥ III; (2) history of previous thoracic surgery; (3) severe dysfunction of major organs; (4) infection at the puncture site or systemic infection; (5) coagulopathy; (6) chronic pain or dependence on opioids, sedatives, antidepressants, or monoamine oxidase inhibitors; (7) history of alcohol abuse; (8) known allergy to dezocine or ropivacaine; and (9) prior chemotherapy or radiotherapy before surgery.

### Sample size estimation

We estimated the sample size for this study using G Power 3.1.9.2 software, with parameters set as Power = 0.90, α = 0.05, Effect size = 0.5, and Number of groups = 2 (N1:N2 = 1), where N1 represents the L-dose group and N2 represents the H-dose group. The minimum required sample size was calculated to be 172, with 86 cases in each group. Subsequently, considering a potential 10% loss of sample size, we ultimately included 192 patients with NSCLC undergoing VATS in this study.

### Randomization process and blinding

A physician A not involved in the study strictly implemented inclusion and exclusion criteria for the overall sample. Before patient inclusion, numbers 001 to 192 were assigned, and 192 random numbers were generated automatically using SPSS software. These random numbers were then matched with corresponding numbers. The random numbers were sorted in ascending order, and those with odd last digits were assigned to the L-dose group, while those with even last digits and zero were assigned to the H-dose group. Physician B, who was not blinded, sealed the grouping results (number, random number, group) along with the corresponding anesthesia protocol in opaque envelopes, which were numbered consistently with the random numbers. Physician C, also not blinded, opened the envelopes in numerical order according to patient enrollment sequence before surgery and implemented the corresponding anesthesia protocol as specified in the envelopes.

This study adopted a double-blind method, with anesthesia and surgical treatment administered according to the allocation scheme to avoid selection bias. All patients were informed that they might receive one of two treatment protocols but were unaware of their specific group assignment. Additionally, personnel responsible for data collection and statistical analysis were also unaware of the group assignments. During the drug preparation phase, all medications were prepared by a pharmacist not involved in anesthesia management. Dezocine was diluted with normal saline to a volume of 10 mL, and the appropriate amount was administered based on the patient’s weight. During the trial, identical syringe models and volumes were used to withdraw the trial drugs. The pharmacist must prepare the drugs in an independent preparation area, place the prepared drugs in containers with consistent appearances, and cover them with treatment drapes. Throughout the drug administration process, the administrator must maintain concealment to prevent the blinded personnel from seeing the drugs, thereby ensuring the validity of the blinding.

### Treatment protocol

All subjects underwent TPVB to complete the surgery. Fifteen minutes before anesthesia induction, the appropriate TPVB puncture point was selected based on the corresponding segment of the planned surgical incision, typically 2–3 cm lateral to the T_4–7_ spinous process on the operative side. Subsequently, the TPVB puncture point was located under ultrasound guidance, and the needle was inserted in-plane into the intervertebral space of the thoracic paravertebral region. After aspiration to confirm the absence of cerebrospinal fluid, blood, or gas, the TPVB drug was injected. The L-dose group received 0.1 mg⋅kg^–1^ dezocine (specification: 1 mL:5 mg, Yangtze River Pharmaceutical Group Co., Ltd., National Medical Products Administration Approval Number H20080329) + 0.375% ropivacaine (specification: 10 mL:50 mg, Ruiyang Pharmaceutical Co., Ltd., National Medical Products Administration Approval Number H20183151), totaling 20 mL; the H-dose group received 0.15 mg⋅kg^–1^ dezocine + 0.375% ropivacaine, also totaling 20 mL. After drug injection, images showing drug diffusion from the puncture point and pleural depression due to pressure could be observed. All subjects received postoperative patient-controlled intravenous analgesia (PCIA) with a protocol of 100 μg sufentanil (specification: 1 mL:75 μg, Yichang Humanwell Pharmaceutical Co., Ltd., National Medical Products Administration Approval Number H20054172) + 0.9% sodium chloride solution, totaling 100 mL, with a pump rate of 2 mL/h, a self-controlled dose of 2 mL, and a single lockout time of 15 min. If the visual analog scale (VAS) score remained > 3 after PCIA activation, a single intravenous injection of 50 mg tramadol injection (specification: 2 mL:100 mg, Shanghai Haifen Pharmaceutical Co., Ltd., National Medical Products Administration Approval Number H20033335) could be administered as analgesic rescue. All patients underwent anesthesia and surgery performed by the same surgical and anesthesia teams.

### Sample collection and data acquisition

Baseline characteristics collected at admission included age, gender, body mass index (BMI), smoking history, comorbidities (diabetes, hypertension), TNM stage, NYHA class, ASA grade, arterial partial pressure of oxygen (PaO2), arterial partial pressure of carbon dioxide (PaCO2), heart rate, and respiratory rate. Surgery and recovery-related variables (surgical duration, time to spontaneous breathing recovery, awakening, and extubation), and the number of analgesic pump uses and the occurrence of analgesic rescue within 12 h postoperatively were recorded. Venous blood samples (5 mL) were collected at three time points—30 min before surgery, 24 h after surgery, and following chest drain removal—for ELISA-based measurement of inflammatory markers.

### Pain assessment

Postoperative pain was evaluated at 1, 6, 12, and 24 h under both resting and active conditions with the VAS, which ranges from 0 (no pain) to 10 (worst pain imaginable) (17). All assessments were performed by the same investigator blinded to group allocation to ensure consistency and minimize bias.

### ELISA assays

Serum inflammatory markers [C-reactive protein (CRP), tumor necrosis factor-α (TNF-α), alongside procalcitonin (PCT)] were measured at three time points: preoperatively, 24 h after surgery, and following chest drain removal. Measurements were performed using ELISA kits (CRP, Cat. No. 1532949572; TNF-α, Cat. No. 1535148262; PCT, Cat. No. 1533145721; all from Shanghai Jianglai Biotechnology, China) according to the manufacturers’ protocols. To ensure consistency and avoid bias, all assays were conducted by the same laboratory physician blinded to group allocation.

### Adverse reactions

Postoperative complications were monitored and recorded, including nausea and vomiting, dizziness or somnolence, pruritus, respiratory depression (defined as a significant reduction in respiratory rate and depth, or even apnea; detailed clinical characteristics and criteria are provided in Supplementary Table 1), and arrhythmias [defined as abnormalities in the frequency or rhythm of cardiac beats, including tachycardia (heart rate > 100 beats/min) and bradycardia (heart rate < 60 beats/min)].

### Statistical analysis

All data were processed and verified independently by two statisticians with over five years of experience, both blinded to treatment allocation. Statistical analyses and figure generation were performed with SPSS software (version 28.0.1.1; SPSS Inc., Chicago, IL, USA) coupled with GraphPad Prism (version 8.0.1; GraphPad Software Inc., San Diego, CA, USA). The Kolmogorov–Smirnov test was employed to assess normal distribution. Measurement data that did not follow a normal distribution were expressed as M (P25, P75), and intergroup comparisons were conducted using the Mann–Whitney U test. For measurement data collected at multiple time points, the Friedman test was utilized. Categorical data between groups were presented as case numbers (percentages), and comparisons were made using the chi-square test (chi-square test/chi-square goodness-of-fit test). The significance level (α) was set at 0.05, with *P*-values representing two-tailed tests. A *P*-value less than 0.05 was considered statistically significant.

## Results

### Baseline characteristics

Baseline demographic and clinical data collected on admission are summarized in Table 1. No differences were noted between the L-dose and H-dose groups in gender, age, BMI, smoking history, diabetes, hypertension, TNM stage, NYHA class, ASA grade, PaO2, PaCO2, heart rate, or respiratory rate (all *P* > 0.05), indicating good comparability between the groups.

**TABLE 1:** Baseline data analysis.

Indicator	L-dose (*n* = 96)	H-dose (*n* = 96)	*z/t/*χ*^2^*	*P*
Gender (male/female)	53/43	59/37	0.771	0.380
Age (years)	53 (50, 56)	52 (50, 55)	1.011	0.312
BMI (kg/m^2^)	23.18 (21.28, 24.87)	23.34 (21.72, 25.02)	0.582	0.561
**Smoking history [case, (%)]**			0.559	0.455
Yes	33 (34.38)	38 (39.58)		
No	63 (65.63)	58 (60.42)		
**Diabetes history [case, (%)]**			0.600	0.439
Yes	18 (18.75)	14 (14.58)		
No	78 (81.25)	82 (85.42)		
**Hypertension history [case, (%)]**			1.380	0.240
Yes	20 (20.83)	27 (28.13)		
No	76 (79.17)	69 (71.88)		
**TNM stage [case, (%)]**			0.858	0.354
I	28 (29.17)	34 (35.42)		
II	68 (70.83)	62 (64.58)		
**NYHA class [case, (%)]**			0.573	0.449
I	60 (62.50)	65 (67.71)		
II	36 (37.50)	31 (32.29)		
**ASA grade [case, (%)]**			0.809	0.368
I	58 (60.42)	64 (66.67)		
II	38 (39.58)	32 (33.33)		
PaO_2_ (mmHg)	90 (84.25, 92)	88 (82, 93)	0.847	0.397
PaCO_2_ (mmHg)	41 (38, 44)	41 (38, 43)	1.049	0.294
Heart rate (bpm)	91 (78.5, 105)	92 (81, 105.75)	0.718	0.472
Respiratory rate (bpm)	23 (20, 26)	22 (20, 25)	0.627	0.531
Surgical duration (min)	184 (167.5, 203.75)	189 (168, 207)	0.509	0.611

BMI, body mass index; TNM, tumor-node-metastasis; NYHA, New York Heart Association; ASA, American Society of Anesthesiologists; PaO_2_, arterial partial pressure of oxygen; PaCO_2_, arterial partial pressure of carbon dioxide. Normality was tested using the Kolmogorov–Smirnov test. Non-normally distributed measurement data were expressed as M (P25, P75) and compared between groups using the Mann–Whitney U test. Categorical data were presented as counts (percentages) and analyzed using the chi-square test (chi-square test/chi-square goodness-of-fit test).

### Surgery and recovery-related times

**FIGURE 2:**
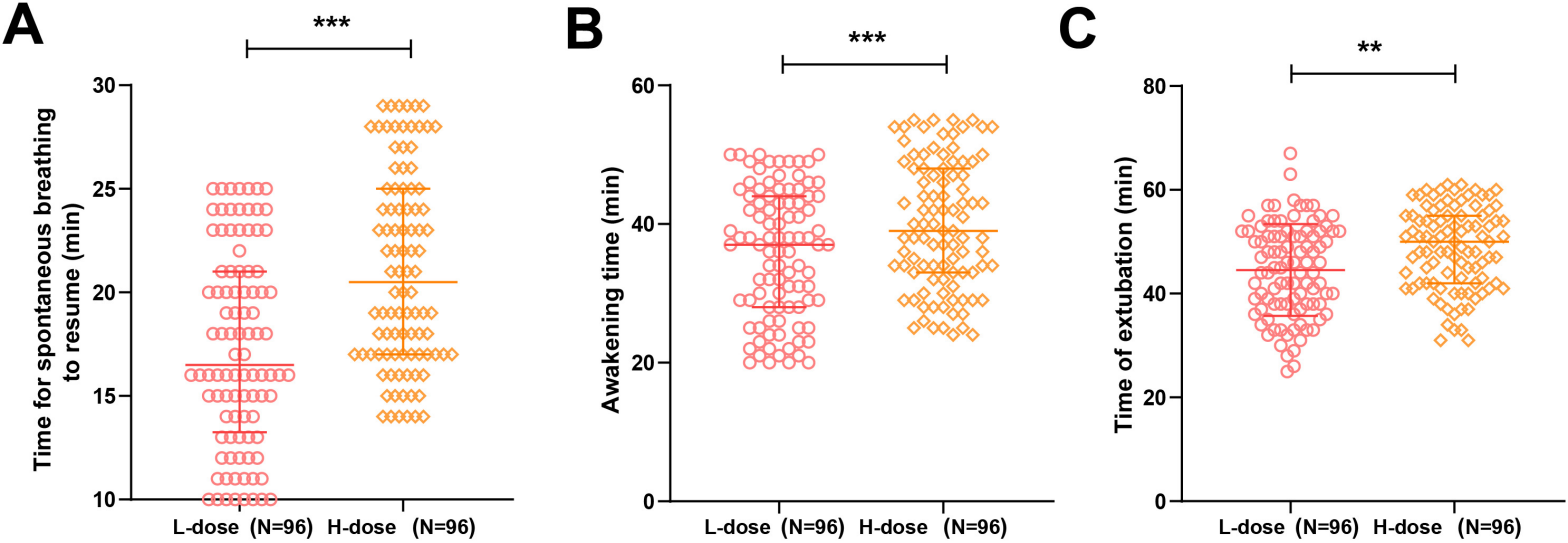
Surgery and recovery-related times. **(A)** Spontaneous breathing recovery time; **(B)** Awakening time; **(C)** Extubation time. The Kolmogorov–Smirnov test was used to assess normal distribution. Measurement data that did not follow a normal distribution were expressed as M (P25, P75), and intergroup comparisons were conducted using the Mann–Whitney U test. For measurement data that followed a normal distribution, the mean ± standard deviation was used for representation, and intergroup comparisons were made using the independent samples *t*-test. ***P < 0.001, **P < 0.01; In **(A,B)**, the error bars represent the interquartile range; In **(C)**, the error bars represent the standard deviation.

Compared with the H-dose group, patients in the L-dose group demonstrated shorter times to spontaneous breathing recovery, awakening, and extubation (all *P* < 0.001) (Figure 2 and Table 2), indicating that, when plus 0.375% ropivacaine, a dose of 0.1 mg⋅kg^–1^ dezocine may facilitate earlier return of spontaneous ventilation and faster readiness for extubation.

**TABLE 2:** Comparison of surgery and recovery-related times between the two groups.

Indicator	L-dose (*n* = 96)	H-dose (*n* = 96)	*z/t*	*P*
Time to spontaneous breathing recovery (min)	16.50 (13.25, 21.00)	20.50 (17, 25)	4.783	< 0.001
Time to awakening (min)	37 (28, 44)	39 (33, 48)	2.639	< 0.001
Time to extubation (min)	44.54 ± 8.86	48.78 ± 7.90	3.501	0.001

The Kolmogorov–Smirnov test was used to assess normal distribution. Non-normally distributed measurement data are presented as M (P25, P75), and comparisons between groups were performed using the Mann–Whitney U test. Normally distributed measurement data are presented as mean ± standard deviation, and comparisons between groups were performed using independent samples *t*-tests.

### Postoperative analgesia

**FIGURE 3:**
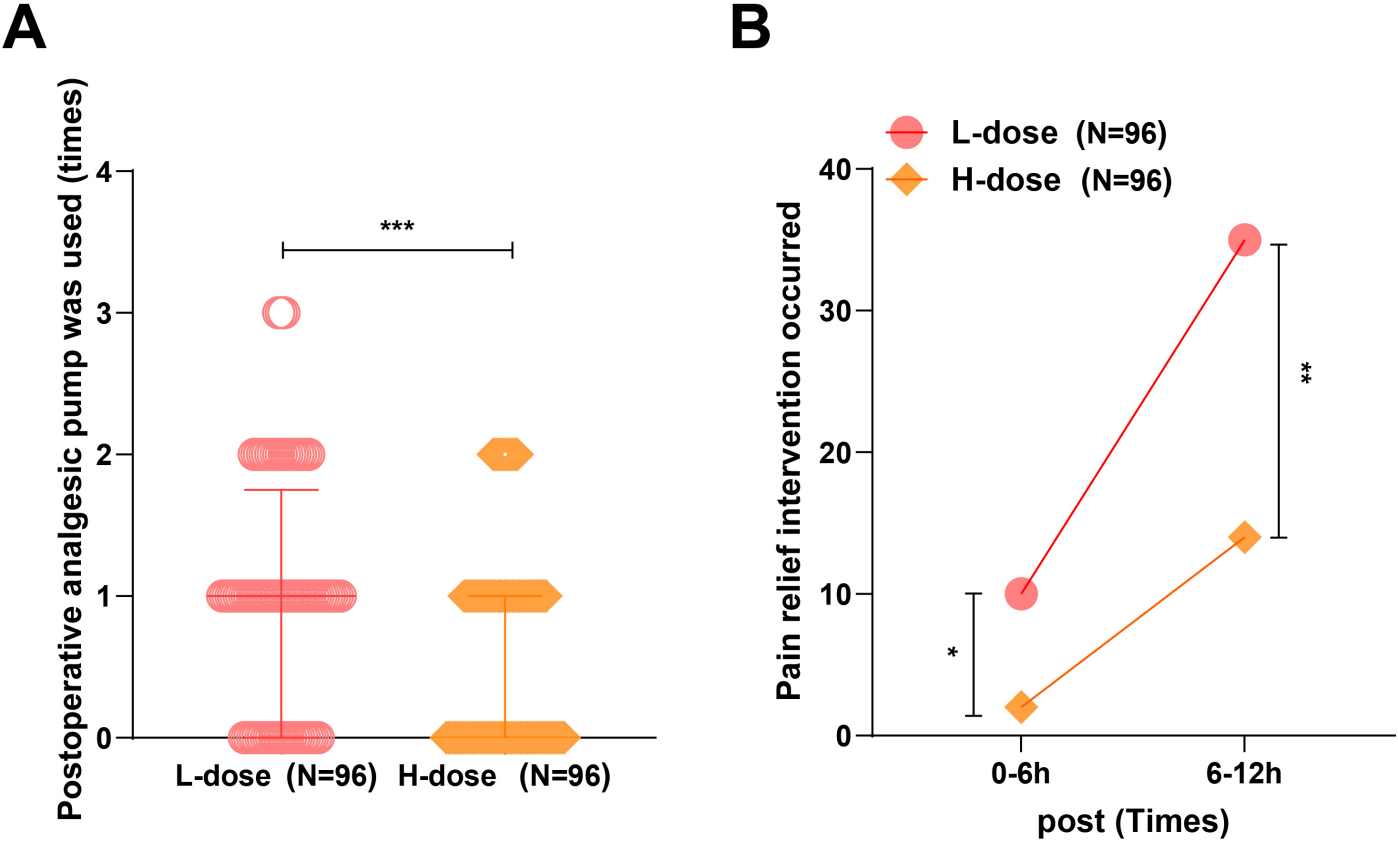
The analgesic pump usage and analgesic rescue within 12 h after surgery. **(A)** Number of analgesic pump uses (measurement data). Normality was tested with the Kolmogorov–Smirnov test. Measurement data that did not follow a normal distribution were expressed as M (P25, P75), and the Mann–Whitney U test was used for between-group comparisons. **(B)** Rescue analgesia (categorical data). Categorical data, presented as *n* (%), and analyzed using the chi-square test for intergroup comparisons. ***P < 0.001, **P < 0.01, *P < 0.05; **(A)** error bar: interquartile range.

In contrast to the L-dose group, patients in the H-dose group presented a significant reduction in analgesic pump uses within 12 h post-surgery (*P* < 0.001). Regarding rescue analgesia, the L-dose group required 10 cases within the first 6 h and 35 cases between 6 and 12 h, whereas the H-dose group required only 2 and 14 cases, respectively. The overall rescue analgesia rate within 12 h was markedly lower in the H-dose group than in the L-dose group (16.67% vs. 46.88%, *P* < 0.001) (Figures 3A, B and Table 3), suggesting that the 0.15 mg⋅kg^–1^ dezocine provided more effective and sustained analgesia within the first 12 h following surgery.

**TABLE 3:** Comparison of analgesic pump usage and analgesic rescue between the two groups within 12 h postoperatively.

Postoperative analgesia	Number of analgesic pump uses within 12 h postoperatively (times)	Number of patients requiring analgesic rescue within 6 h postoperatively [*n* (%)]	Number of patients requiring analgesic rescue between 6 and 12 h postoperatively [*n* (%)]	Total analgesic rescue rate within 12 h postoperatively [*n* (%)]
L-dose (*n* = 96)	1 (0, 1.75)	10 (10.42)	35 (36.46)	45 (46.88)
H-dose (*n* = 96)	0 (0, 1)	2 (2.08)	14 (14.58)	16 (16.67)
*z/*χ*^2^*	3.505	5.689	12.080	20.210
*P*	< 0.001	0.012	0.001	< 0.001

The Kolmogorov–Smirnov test was used to assess normal distribution. Measurement data that did not follow a normal distribution were expressed as M (P25, P75), and intergroup comparisons were conducted using the Mann–Whitney U test. Categorical data, presented as *n* (%), and analyzed using the chi-square test for intergroup comparisons.

### Pain assessment at different time points

**FIGURE 4:**
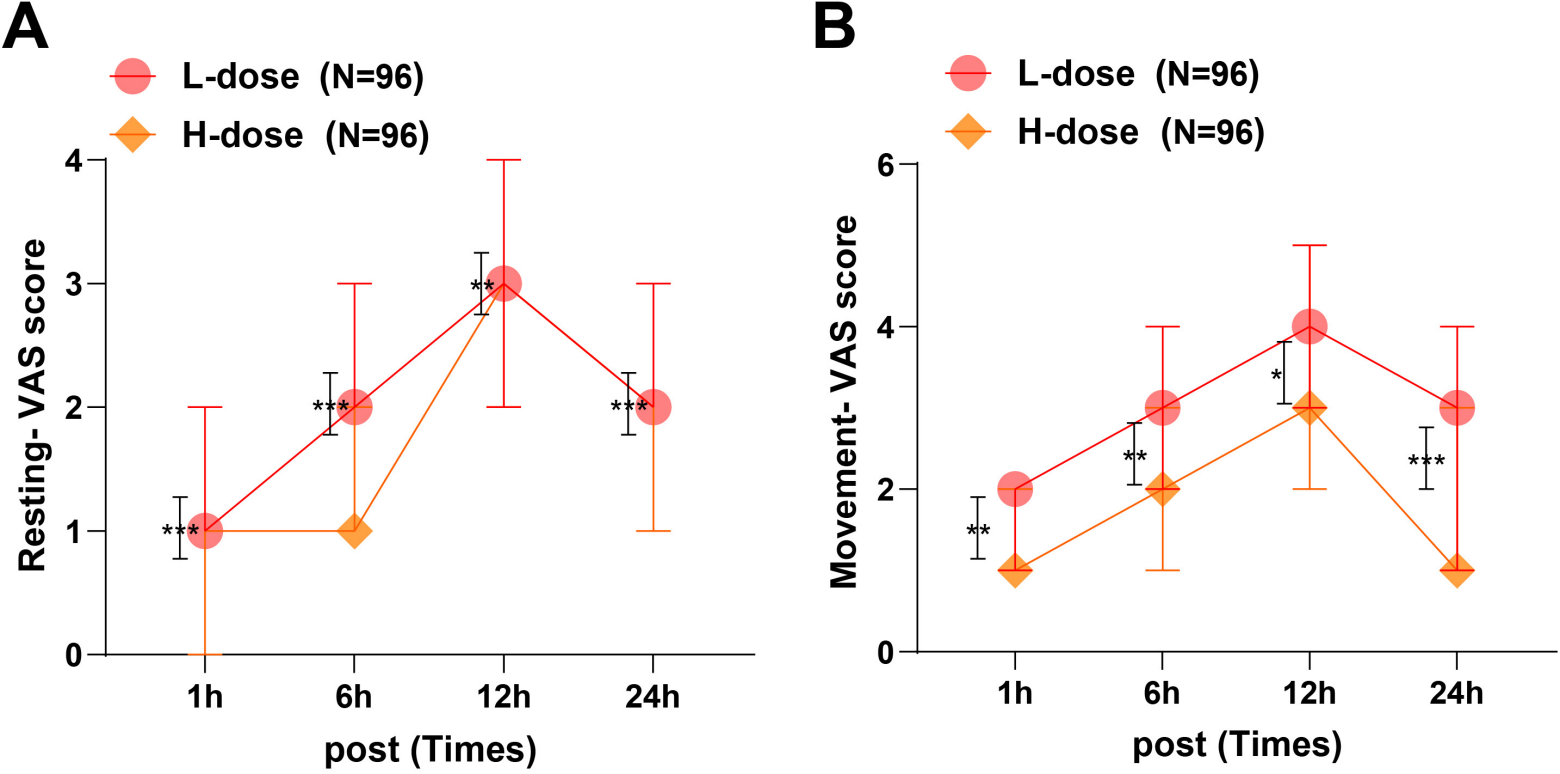
Pain conditions at different stages and states. **(A)** Comparison of VAS scores within and between groups at different time points in the resting state; **(B)** Comparison of VAS scores within and between groups at different time points in the moving state. The Kolmogorov–Smirnov test was performed for normality assessment. Measurement data that did not follow a normal distribution were expressed as M (P25, P75). Group comparisons were conducted using the Mann–Whitney U test, while repeated measures at 1, 6, 12, and 24 h were analyzed with the Friedman test. *P < 0.05, **P < 0.01, ***P < 0.001; error bar: interquartile range.

In both groups, VAS scores at rest and during movement increased progressively with postoperative recovery and then declined at 24 h (all *P* < 0.05). Notably, at every time point and under both conditions, VAS scores in the H-dose group were significantly lower than the L-dose group (all *P* < 0.001) (Figure 4 and Table 4). This confirmed that the 0.15 mg⋅kg^–1^ dezocine offered superior pain relief and longer-lasting analgesic effects during the first 24 h postoperatively.

**TABLE 4:** Comparison of pain scores between the two groups at different stages and states.

Pain scores	VAS score at rest (points)				VAS score during movement (points)			
	1 h postoperatively	6 h postoperatively	12 h postoperatively	24 h postoperatively	1 h postoperatively	6 h postoperatively	12 h postoperatively	24 h postoperatively
L-dose (*n* = 96)	1 (1, 2)	2 (2, 3)	3 (2, 4)	2 (2, 3)	2 (1, 2)	3 (2, 4)	4 (3, 5)	3 (1, 4)
H-dose (*n* = 96)	1 (0, 1)	1 (1, 2)	3 (2, 3)	2 (1, 2)	1 (1, 2)	2 (1, 3)	3 (2, 5)	1 (1, 3)
*Z*	4.997	4.321	3.192	3.859	3.142	3.173	2.506	3.931
*P*	< 0.001	< 0.001	0.001	< 0.001	0.002	0.002	0.012	< 0.001

VAS, visual analog scale. The Kolmogorov–Smirnov test was used to assess normal distribution. Non-normally distributed measurement data are presented as M (P25, P75), and comparisons between groups were performed using the Mann–Whitney U test. Measurement data at 1, 6, 12, and 24 h postoperatively were tested using the Friedman test.

### Serum inflammatory factor levels

**FIGURE 5:**
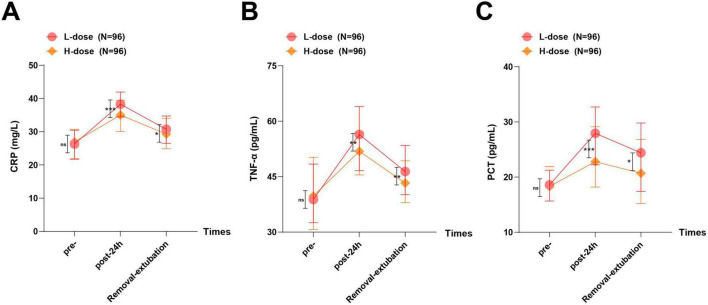
Serum inflammatory factor levels at different stages. **(A)** Comparison of CRP within and between groups at different time points; **(B)** Comparison of TNF-α within and between groups at different time points; **(C)** Comparison of PCT within and between groups at different time points. Normality was evaluated with the Kolmogorov–Smirnov test. Non-normally distributed measurement data were presented as M (P25, P75). The Mann–Whitney U test was applied for intergroup comparisons, while repeated measures preoperatively, 24 h postoperatively, and after chest drainage tube removal (Removal-extubation) were analyzed with the Friedman test. ns: *P > 0.05, **P < 0.01, ***P < 0.001; error bar: interquartile range.

As presented in Figure 5 and Table 5, there were no significant differences in preoperative levels of CRP, TNF-α, and PCT between the two groups (all *P* > 0.05). Compared with preoperative levels, serum CRP, TNF-α, and PCT levels in both groups showed a trend of initial increase followed by decrease at 24 h postoperatively and at removal-extubation, and serum CRP, TNF-α, and PCT levels in the H-dose group were lower than those in the L-dose group at 24 h postoperatively and at removal-extubation, suggesting that 0.15 mg⋅kg^–1^ dezocine was more effective in mitigating surgery-induced inflammatory responses when combined with ropivacaine.

**TABLE 5:** Comparison of serum inflammatory factor levels between the two groups at different stages.

Serum inflammatory factor levels	CRP (mg/L)			TNF-α (pg/mL)			PCT (pg/mL)		
	Preoperatively	24 h postoperatively	Removal-extubation	Preoperatively	24 h postoperatively	Removal-extubation	Preoperatively	24 h postoperatively	Removal-extubation
L-dose (*n* = 96)	26.32 (21.72, 30.44)	38.29 (34.43, 41.93)	30.73 (26.50, 34.74)	38.83 (32.56, 48.40)	56.42 (46.61, 63.99)	46.39 (40.12, 53.42)	18.60 (15.66, 21.25)	27.94 (22.24, 32.72)	24.41 (17.42, 29.80)
H-dose (*n* = 96)	26.92 (21.85, 30.67)	35.01 (30.07, 39.47)	29.30 (24.87, 33.96)	39.74 (30.71, 50.24)	51.87 (45.49, 56.89)	43.28 (37.99, 49.28)	18.32 (14.10, 21.90)	22.80 (18.19, 29.13)	20.69 (15.23, 26.84)
*z*	0.026	4.523	2.332	0.17	2.644	2.745	0.249	4.133	2.527
*P*	0.979	< 0.001	0.02	0.865	0.008	0.006	0.803	< 0.001	0.011

CRP, C-reactive protein; TNF-α, tumor necrosis factor-α; PCT, procalcitonin. Normality was evaluated with the Kolmogorov–Smirnov test. Non-normally distributed measurement data were presented as M (P25, P75). The Mann–Whitney U test was applied for intergroup comparisons, while repeated measures preoperatively, 24 h postoperatively, and after chest drainage tube removal (Removal-extubation) were analyzed with the Friedman test.

### Adverse events

The overall incidence of adverse events is summarized in Table 6. Reported events included nausea, vomiting, dizziness, drowsiness, pruritus, respiratory depression, and arrhythmia. The overall incidence of adverse reactions was 8.33% in the H-dose group and 6.25% in the L-dose group, with the H-dose group being slightly higher than the L-dose group; however, statistical analysis showed no significant difference (*P* > 0.05). All adverse reactions were mild and returned to normal after intervention. Interventions for mild respiratory depression included adjusting the patient’s position, clearing oral and nasal secretions to maintain airway patency, providing assisted ventilation via a face mask, and closely monitoring the patient’s vital signs. These findings indicated that compared to a dose of 0.1 mg⋅kg^–1^ dezocine, a dose of 0.15 mg⋅kg^–1^ did not significantly increase the risk of adverse reactions and exhibited relatively higher safety.

**TABLE 6:** Adverse reaction occurrences.

Adverse reaction	Nausea and vomiting	Dizziness and drowsiness	Pruritus	Respiratory depression	Arrhythmia	Total adverse reactions
L-dose (*n* = 96)	1 (1.04)	3 (3.13)	1 (1.04)	0 (0.00)	1 (1.04)	6 (6.25)
H-dose (*n* = 96)	3 (3.13)	2 (2.08)	1 (1.04)	2 (2.08)	0 (0.00)	8 (8.33)
χ*^2^*	–					0.308
*P*	–					0.579

Categorical variables were presented as counts (percentages) and analyzed using the chi-square test (chi-square test/chi-square goodness-of-fit test).

## Discussion

Surgical resection remains the gold standard for treating early-stage lung cancer, including traditional thoracotomy and minimally invasive surgery using VATS (18). Over the past two decades, the development of VATS technology has revolutionized general thoracic surgery, offering patients numerous benefits such as reduced postoperative complications, decreased postoperative pain, shorter hospital stays, and improved postoperative pulmonary function, with long-term survival rates comparable to those of thoracotomy (18). However, evidence confirms that it can trigger inflammatory responses, and the degree of inflammation is closely related to the quality of postoperative recovery (7). This study compared the effects of two different doses of dezocine (0.1 mg⋅kg^–1^ vs. 0.15 mg⋅kg^–1^) combined with 0.375% ropivacaine for TPVB in VATS for lung cancer. The results indicated that in patients undergoing VATS for lung cancer, the TPVB regimen combining dezocine with ropivacaine yielded differentiated short-term benefits, with the two doses offering distinct advantages: 0.1 mg⋅kg^–1^ dezocine promoted faster postoperative awakening, while 0.15 mg⋅kg^–1^ dezocine provided superior analgesia and inflammation suppression.

Ropivacaine is widely applied in regional anesthesia due to its prolonged action and relatively low systemic and neurotoxicity. However, its analgesic effect alone may be insufficient, prompting investigations into adjuvant combinations to enhance efficacy (19). It has been reported that serratus anterior plane block with 0.5% ropivacaine plus dexmedetomidine (1 μg/kg) combined with patient-controlled intravenous analgesia significantly reduced postoperative pain and improved recovery in VATS patients (20). Another report states that for patients undergoing VATS, intraoperative intravenous ketamine successfully reduces acute postoperative pain, but it does not exert a significant impact on opioid use, hemodynamic parameters, and the occurrence of adverse events (21). These studies suggest that ropivacaine benefits from adjuncts to achieve optimal outcomes.

This study found that compared to patients in the H-dose group, those in the L-dose group had shorter recovery times for spontaneous breathing, awakening, and extubation. This may suggest that low-dose dezocine (0.1 mg⋅kg^–1^) combined with ropivacaine has certain advantages in promoting early postoperative recovery in patients. Additionally, patients in the H-dose group had lower analgesic pump uses and rescue rates within 12 h postoperatively, lower VAS scores at rest and during movement at various time points, and lower serum CRP, TNF-α, and PCT levels at 24 h postoperatively and at extubation, suggesting that dezocine may enhance analgesia and suppress systemic inflammatory responses in a dose-dependent manner when used in combination, with high-dose dezocine (0.15 mg⋅kg^–1^) demonstrating more significant advantages in postoperative analgesia and inflammation regulation.

In terms of pain relief mechanisms, ropivacaine, as a long-acting amide local anesthetic, produces analgesic effects through local nerve block. At lower concentrations, it can achieve differential blockade of sensory and motor nerves, effectively blocking pain signal transmission while maximizing the preservation of motor function (22, 23). Dezocine, as a mixed opioid receptor agonist-antagonist, primarily activates κ receptors (inhibiting pain signal transmission) and partially activates μ receptors (reducing side effects such as respiratory depression associated with traditional opioids), while enhancing analgesic effects by inhibiting norepinephrine reuptake (12, 24). Prior studies have confirmed its utility both as a primary analgesic and as part of balanced anesthesia regimens (13, 14). Clinical research further demonstrates that combining dezocine with propofol improves perioperative recovery and reduces pain during laparoscopic procedures (25).

In terms of anti-inflammatory mechanisms, ropivacaine and dezocine play synergistic roles in anti-inflammatory effects. Ropivacaine may reduce the production of pro-inflammatory factors such as TNF-α and IL-6 by inhibiting inflammatory signaling pathways such as NF-κB (23). For example, animal experiments by Zhang et al. (23) showed that ropivacaine can inhibit neuroinflammation. Dezocine may regulate immune cell function (13, 26) and reduce the release of inflammatory mediators by activating κ receptors and inhibiting norepinephrine reuptake (12, 24). Additionally, its non-opioid receptor mechanisms (12, 24) may further intervene in the inflammatory process. One study indicated that anesthesia with dezocine plus dexmedetomidine has been shown to attenuate inflammatory responses, preserve brain function, and improve postoperative cognitive outcomes in lung cancer surgery patients (15).

Notably, dezocine combined with ropivacaine infiltration has been shown to reduce stress responses, stabilize immune function, and lower adverse reaction rates, thereby aiding recovery (7). Consistent with this, our study demonstrated that the overall incidence of adverse reactions was slightly higher in the H-dose group than in the L-dose group, but statistical analysis revealed no significant difference. This suggests that within the dose range studied, high-dose dezocine combined with ropivacaine did not significantly increase the risk of adverse reactions, demonstrating a certain degree of safety.

However, this study has several limitations. First, no control group using ropivacaine alone was included, making it impossible to clearly determine the independent contribution of dezocine in the combined regimen and its additional clinical value compared to ropivacaine monotherapy. Second, the study population was limited to relatively young patients with lower ASA grades and earlier tumor stages, restricting the generalization of conclusions to older patients, those with multiple comorbidities, or those with advanced lung cancer. Additionally, the observation indicators focused on the first 24 h postoperatively, lacking an assessment of medium- to long-term recovery, making it difficult to fully reflect the sustained benefits of different dose regimens. We also did not include a placebo control group or analyze the impact of different surgical approaches on the results, posing challenges to the completeness and generalizability of the study conclusions.

In conclusion, both dezocine regimens in combination with ropivacaine demonstrated clinical value during the short-term postoperative period following VATS for NSCLC: the lower dose (0.1 mg⋅g^1^) is advantageous for rapid awakening and ventilator weaning, while the higher dose (0.15 mg⋅kg^–1^) is superior for sustained analgesia, anti-inflammatory effects, and reduced reliance on rescue analgesia, without compromising safety. This provides preliminary clinical references for selecting anesthesia regimens. Future studies should involve larger sample sizes, multiple control groups (including ropivacaine monotherapy and placebo groups), a broader population, and longer follow-up periods to further validate the anti-inflammatory and analgesic mechanisms and clarify their positioning in the comprehensive perioperative management of lung cancer.

## Data Availability

The raw data supporting the conclusions of this article will be made available by the authors, without undue reservation.
