# Supplementary material for: Application and safety analysis of paravertebral block with dezocine and ropivacaine in video-assisted thoracoscopic surgery for lung cancer
Source: Front Med (Lausanne). 2026 Jan 27;12:1723385. doi: 10.3389/fmed.2025.1723385 (PMC12886470; doi:10.3389/fmed.2025.1723385)
Supplement: Supplementary file 1 [file Table_1.docx]

**Supplementary Table 1** Clinical characteristics of respiratory depression

| Clinical characteristic | Diagnostic criteria |
| --- | --- |
| Respiratory rate | Significantly decreased (< 10 breaths/min) or apnea |
| Tidal volume | Significantly reduced (< 5 mL/kg) |
| Arterial oxygen saturation | < 90% |
| Arterial partial pressure of oxygen | < 60mmHg |
| Arterial partial pressure of carbon dioxide | > 50mmHg |
| Clinical manifestations | Presence of cyanosis, labored breathing, confusion, etc. |
